# Microfilariae infestation of goliath frogs (*Conraua goliath*) from Cameroon

**DOI:** 10.1371/journal.pone.0217539

**Published:** 2019-05-29

**Authors:** Daniel Nguete Nguiffo, Charles S. Wondji, Josué Pone Wabo, Mbida Mpoame

**Affiliations:** 1 Research Unit of Biology and Applied Ecology, Department of Animal Biology, Faculty of Science, University of Dschang, Dschang, Cameroon; 2 Centre for Research in Infectious Diseases, Yaoundé, Cameroon; 3 Vector Biology Department, Liverpool School of Tropical Medicine, Liverpool, United Kingdom; Vanderbilt University School of Medicine, UNITED STATES

## Abstract

The goliath frog (*Conraua goliath*) is endemic to Equatorial Guinea and Cameroon. It is an endangered species but little information is known about its parasites. To understand the impact of blood parasites on this species, we microscopically examined blood smears from 78 goliath frogs in February and November 2016 (dry and wet seasons) from six localities in Littoral Region of Cameroon, and we sequenced mitochondrial DNA from positive samples. Microfilariae were found in 33/78 (42.3%) goliath frogs at six locations. No other haemoparasite species was detected. Morphological characteristics of microfilariae were also described, and specimens from each frog species were similar. DNA sequencing data from the mitochondrial Cytochrome Oxidases sub unit I (COI) gene revealed a close relationship with *Icosiella neglecta*, a microfilaria documented in other European, Asian, and African frogs. However, sequences were sufficiently genetically distant (0.118) that they may define a new species of *Icosiella*. The infection burden of microfilariae varied by site, with season (65% in dry season to 23% in rainy season), and by sex, (male frogs had significantly higher parasite burdens than females (p < 0.0001)). However, this may have been confounded by size as the microfilaria intensity increased with frog weight (p < 0.0001), and males were larger than females. Microfilaria infection intensity varied from 1 to 120 per 50 μl of blood. Microfilaria induced a significant increase (p < 0.05) in the number of white blood cells (WBC) counted compared to uninfected frogs, but there was no statistically significant variation in red blood cell (RBC) count, plasma cholesterol level (p = 0.210) or plasma glucose level (p = 0.100).

## Introduction

Amphibian populations have been declining at an alarming rate in many regions of the world due to habitat loss and fragmentation, pollution, climate change and emerging infectious diseases [1–3]. The goliath frog is the largest known frog, weighing up to 3.3 kg and can live up to 15 years in captivity [4]. They are found in or near the waterfalls in parts of Cameroon and Equatorial Guinea. Their habitat is marked by two seasons, the dry season (November to April) and rainy season (May to October) [5]. Goliath frog populations have declined by more than half during the last decade mainly due to deforestation and overhunting [4]. They are classified as “Endangered” by the International Union of Conservation Nature (IUCN) and animals of class “A”, prohibited to hunting without authorisation by fauna Cameroonian law. Today, very little information is available on the ecology and the parasites of this frog [6]. Amphibians are known to carry several hematophagous vectors in their aquatic and terrestrial habitats which infect them with a wide variety of intra- and extra-cellular blood parasites, including viruses, rickettsiae, species of several genera of protozoa, yeast, and microfilariae [7–10]. Among these haemoparasites, Filarioidea nematodes are typically found in the blood and the lymphatic system [11]. The spread of microfilariae out of the lymphatic system causes lethargy, and certain filarial species such as *Foleyella* sp. and *Waltonella* sp. cause mortality in frogs with heavy infections [12]. The aim of this study was to investigate the potential role of blood-borne parasites on goliath frog health. We specifically searched and characterized the haemoparasites found in the goliath frog’s blood in relation to habitat, seasonality, and gender, and we evaluated the haematology and biochemical changes in frogs naturally infected with these parasites.

## Materials and methods

### Ethics statement

This study was approved by the Ministry of Forestry and Wildlife (MINFOF) under reference number of 5188/L/MINFOF/SG/DFAP/SDCF/SEP/ESH and covered the entire study area. The animals used in this study were not sacrificed and all were released alive at the sampling site after blood collection.

### Study area and frog sampling

Our study was conducted in February and November 2016 during the dry and rainy seasons in six localities (Table 1) in the Littoral Region of Cameroon. A total of 78 goliath frogs were captured by hand and fishing. A blood sample was collected from each frog by cardiac puncture [13, 14], stored in an EDTA tube. One drop (around 50 μl) was placed on a slide and a smear made.

**Table 1:** Sampling site area.

Sampling site	GPS coordinates	Number of frogs collected	Land Use
**Nkebe**	04°41’58.7” N 010°08’22.4’ E	14	Forest
**Bantoum**	04°42’56.3” N 010°12’01.9” E	17	
**Bipelhe**	04°43’08,6”N 009°50’42,8” E	15	Agricultural
**Gounja**	04°42’03” N 009°46’14,8” E	13	
**Mbete**	04°41’07,4” N 009°44’55,9” E	11	
**Mpoula**	04°38’38,1” N 009°43’03,3” E	08	

### Slide analysis

Blood smear slides from the 78 goliath frogs were examined under the microscope (100X magnification) for large haemoparasites like *Trypanosoma* and microfilariae (approximately 5 minutes per slide per magnification). For slides that tested positive for microfilariae, the entire slide was examined at 100X, and the number of microfilariae recorded as intensity (i.e. total number per 50 μl of blood). All fields were also observed at 1000X oil-immersion to screen for smaller haemoparasites, e.g., *Plasmodium* and *Haemoproteus*.

### Haematological and biochemical analysis

The blood in EDTA tubes was used for Red Blood Cell (RBC) and White Blood Cell (WBC) counts following the classical method, using the Neubauer hematocymeter [15]. The glucose and cholesterol levels were analysed using CHRONOLAB protocol [16].

### Filaroidae genotyping and phylogenetic analysis

DNA samples were extracted from the blood and preserved in EDTA (Livak, 1989). From these samples, blood DNA from 3 goliath frogs that tested positive for microfilariae from visual inspection were amplified using primers designed specifically for filarial nematodes [17]. We amplified a 601 bp region of the mitochondrial cytochrome *c* oxidase subunit I gene (COIintF 5’-TGATTGGTGGTTTTGGTAA-3’ and COIintR 5’-ATAAGTACGAGTATCAATATC-3’). This locus has been shown to reliably diagnose nematode species [18]. Each Polymerase Chain Reaction (PCR) tube contained 15μl of a PCR master mix of the following components: 1.5 μl PCR buffer A, 0.75 μl 25mM MgCl_2_, 0.12 μl 25mM dNTPs, 0.51 μl forward and reverse primers 10mM, 0.12 μl Kapa Taq, 10.49 μl ddH_2_O and 1 μl DNA. PCR thermocycler conditions were: 5min at 95°C followed by 35 cycles of denaturing at 94°C for 30s, annealing at 48°C for 30s and extension at 72°C for 45s, finishing with an extension step at 72°C for 10min. Amplification products were detected by using 1.5% agarose gel stained with Midori green and electrophoresed. The bands were visualized under UV light and PCR products were then purified using Exo Sap kit according to the manufacturer’s instructions and sent to a company (GENEWIZ, United Kingdom) for Sanger sequencing.

### Statistical analysis

Haematological and biochemical results were expressed as mean ± standard deviation (SD). Values from healthy versus infected groups were compared by the Student’s t-test using GraphPad prism version 5. The Generalized linear mixed model was built and run with 'lme4' package [19] in R version 3.4 to evaluate the influence of habitat, season, host, weight and gender on microfilaria intensity. The significance level was calculated using the Bonferroni correction.

### Phylogenetic analysis

The chromatogram (derived from Sanger sequencing using dideoxynucleotides with fluorescence-tags) was checked manually (GenBank: MH182623) using BioEdit 5.0. The nucleotide sequences of individual genes and the sequences of various genes taken from GenBank were initially aligned using BioEdit 5.0 [20] with Clustal W program and then manually aligned. Phylogenetic trees were generated using *MEGA* version 7.0.26 by Maximum Likelihood (ML) method with (General Time Reversible best model) based on the Tamura Nei model [21] with 1000 replicates. The genetic distance (Tamura Nei model) between the microfilaria was estimated using MEGA version 6.06.

## Results

### Prevalence and microfilaria intensity variation between the seasons and habitat

Across the 2 sampling periods (February and November 2016), the prevalence of microfilariae was reduced significantly (p < 0.001) from 65.71% (23/35) during the dry season to 23.25% (12/43) during the wet season. The agricultural settings were not significantly different from forest areas with a prevalence of 36% (23/47) vs 48% (15/31) respectively.

The intensity of infection in individual frogs ranged from 1–120 per slide (using 50μl of blood). The mean intensity per millilitre of blood was significantly (p < 0.001) different between seasons (323/ml in the dry season vs 86/ml in the wet season). Intensity also correlated with body weight and sex with an increase in intensity of infection in relation to weight (p < 0.001) and sex with the males being more heavily infected than females (p < 0.001) (Table 2). However, the observed sex differences may have been confounded by weight, as males were larger than females (mean weight 1489.5 g/male vs 745 g/female).

**Table 2:** Influence of Land use, weight, gender of goliath frogs and season in relation to the microfilariae intensity.

	Estimation	Standard error	Z value	p value
**Intercept**	5,43055	0,48660	10,44***	< 1.6^e-15^
**Weight**	0,44464	0,03598	12,36***	< 1.6^e-15^
**Season**	-2,42860	0,03888	-62,40***	< 1.6^e-15^
**Gender**	-0,25400	0,03472	-7,32 ***	2,4^e-5^
**Land use**	-0,82541	0,09895	-1,31	0.231

*** p < 0.0001

### Influence of microfilarial infestation on haematological and biochemical parameters

**Fig 1:**
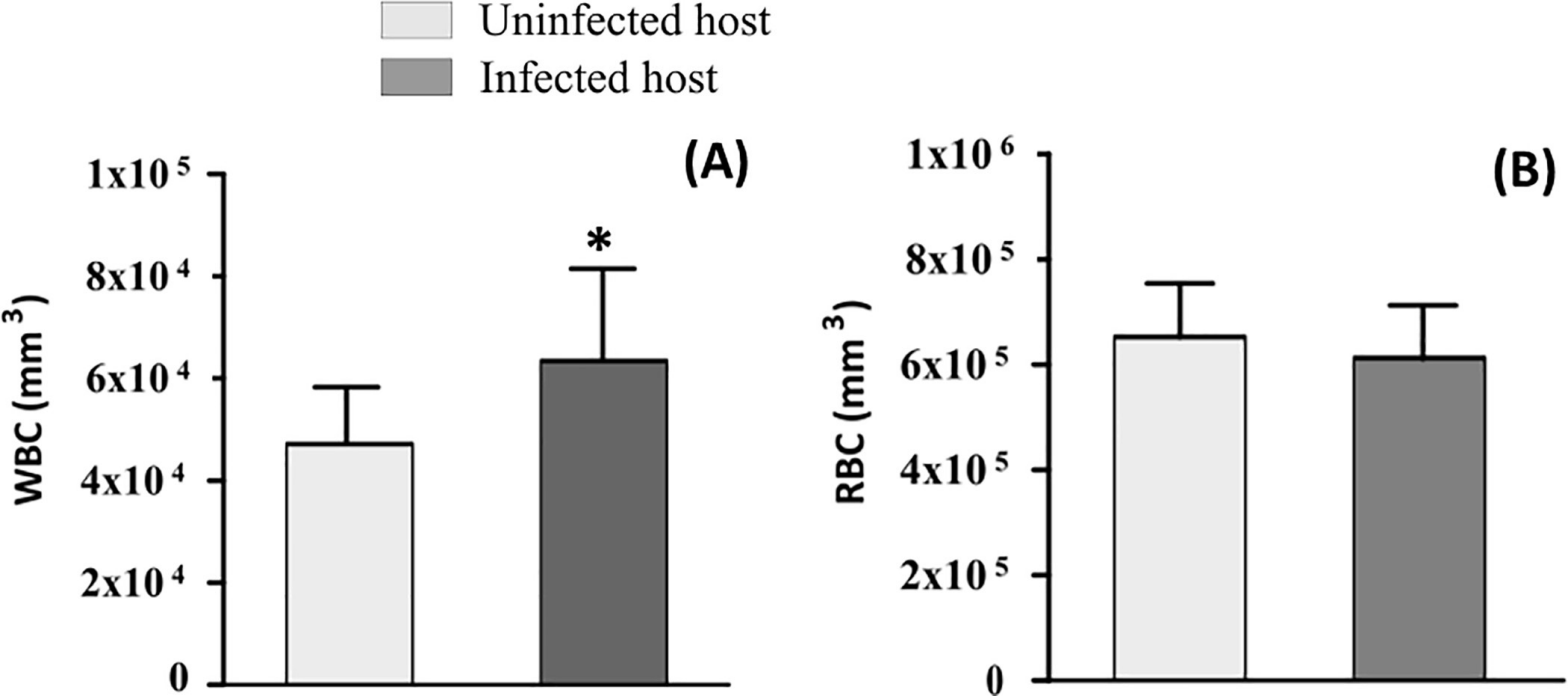
White Blood Cell counts (WBC) (A) and Red Blood Cell counts (RBC) (B) in healthy vs infected frogs.

**Fig 2:**
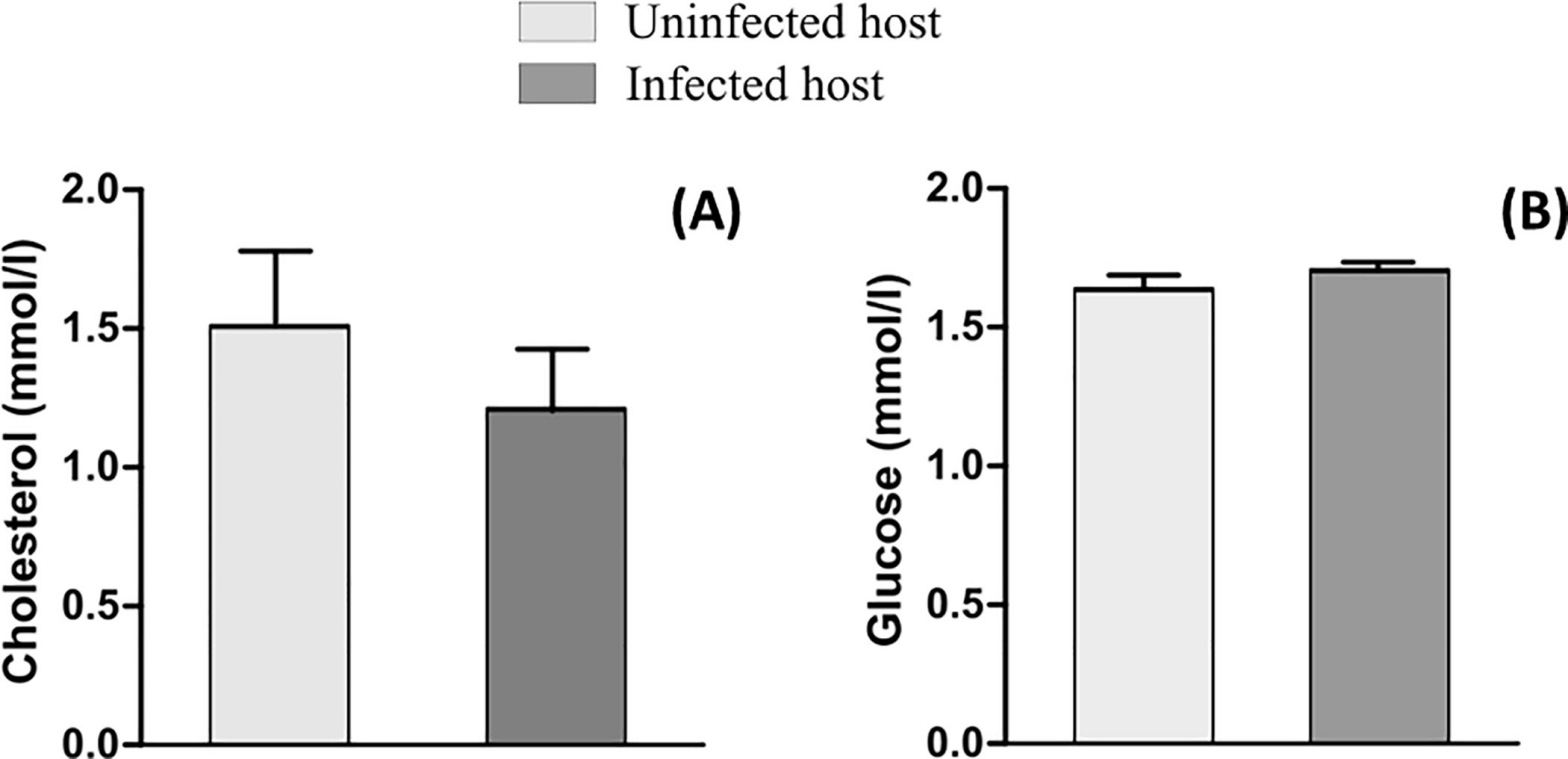
Plasma glucose (A) and Cholesterol (B) in healthy vs infected frogs.

The effects of microfilaria infection on haematological parameters in frogs are shown in Fig 1. In comparison with healthy frogs, microfilaria infection induced a significant increase in WBC count (p<0.05) and no variation in RBC count (p>0.05) in frogs. Biochemical plasma values are shown in Fig 2 where the value of plasma cholesterol levels (p = 0.210) and plasma glucose levels (p = 0.100) were not significantly different in infected compared to non-infected individuals.

### Filaroidae genotyping and phylogenetic analyses

**Fig 3:**
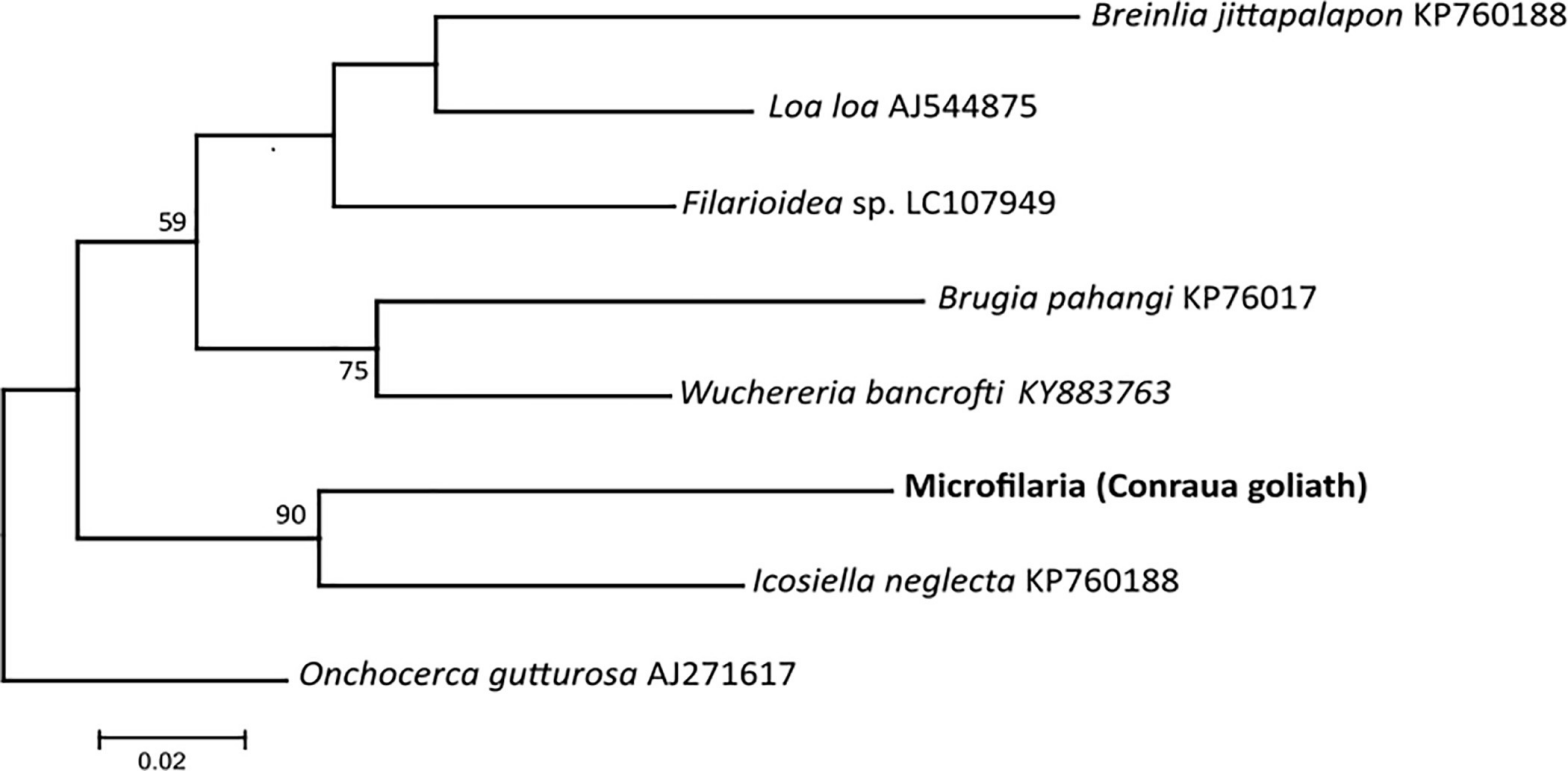
Phylogenetic tree from the mitochondrial cytochrome *c* oxidase subunit I gene of filarid nematodes, including sequences obtained from goliath frog microfilariae generated by maximum-likelihood conducted in MEGA. The bootstrap values below 50 have been removed on the node.

A 601-bp region of double-stranded filarial nematode mitochondrial COI sequence was recovered from DNA recovered using extraction and PCR from 3 heavily infected goliath frogs initially screened using a haemocytometer. Filarial COI sequences from these 3 individuals were identical. We subjected these filarial COI sequences to a BLAST search in GenBank (NCBI). All of the most similar sequences in the database were filarial nematode COI sequences, and the 3 goliath frog derived sequences were close to *Icosiella neglecta*, microfilaria of European, and Asia amphibians (Fig 3). The genetic distance between different sequences shows goliath frog microfilaria closest to *Icosiella neglecta* with a value of 0.118 (Table 3).

**Table 3:** Genetic distance (Tamura Nei model) between goliath frog microfilaria and another filarial species found in GenBank.

Species	*Conraua goliath* Microfilariae	*Icosiella neglecta*	*Wuchereria bancrofti*	*Onchocerca* *gutturosa*	Filarioidea sp	*Loa loa *
***Icosiella neglecta***	0.118					
***Wuchereria bancrofti***	0.126	0.126				
***Onchocerca*** ***gutturosa***	0.131	0.116	0.104			
***Filarioidea* sp. **	0.136	0.126	0.105	0.104		
***Loa loa ***	0.126	0.139	0.115	0.111	0.084	
***Breinlia jittapalapon***	0.145	0.160	0.128	0.114	0.114	0.113

### Microfilariae morphology

**Fig 4:**
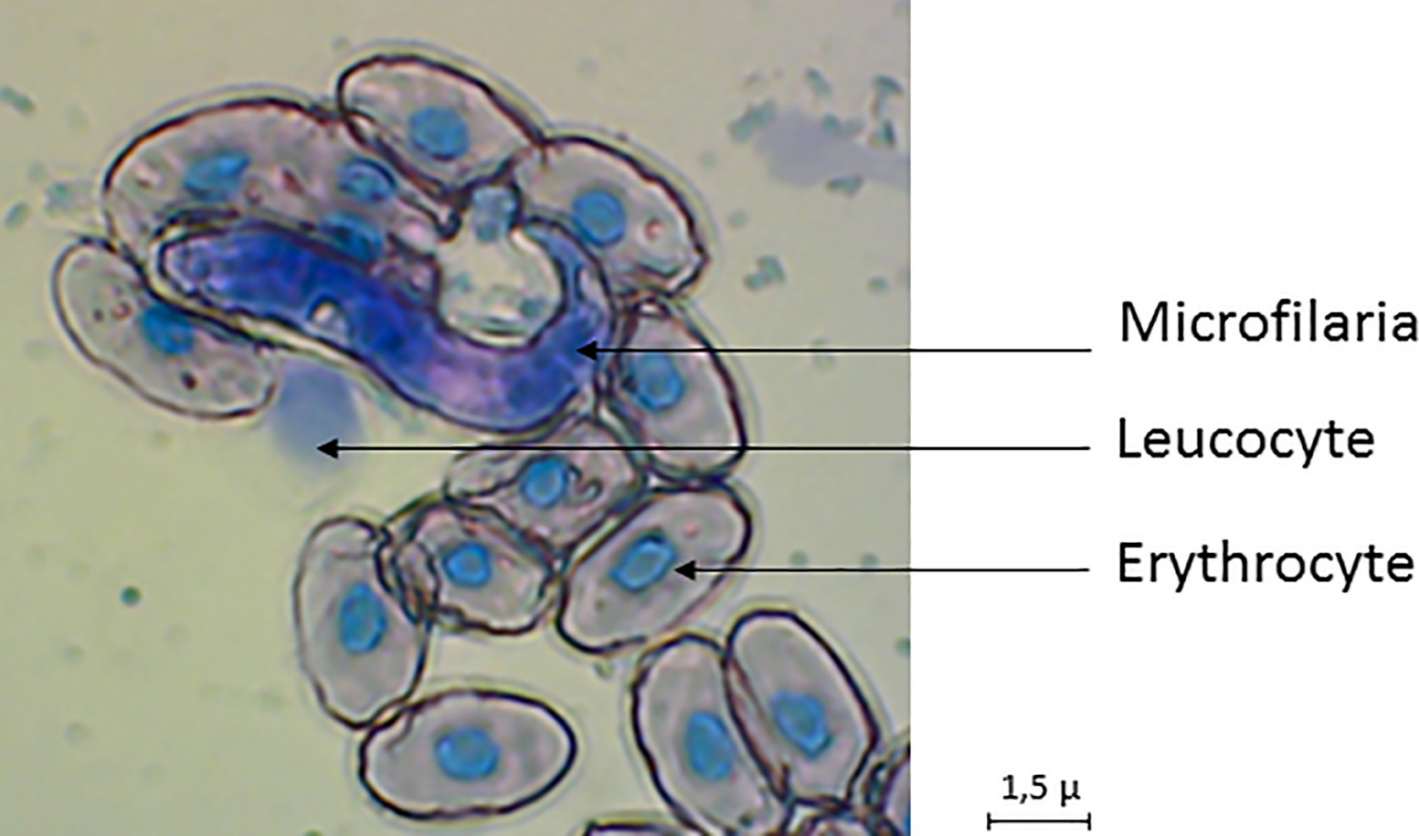
Picture of goliath frog microfilariae.

The microfilariae from goliath frogs are relatively short with mean length of 64.8μm and 0.75μm in width. The nucleus is extended over the entire body except for cephalic space which measures approximately 1.2μm and at 70% of length of body (Fig 4). Posterior end is sharp and seen covered with nucleus after coloration. Microfilariae from *C*. *goliath* were similar in appearance and measurement to *Icosiella* genus members. This filaria may represent a new species.

## Discussion

This work is the first to assess goliath frog blood parasites and reveals that they are primarily affected by one species of microfilaria belonging to the *Icoseilla* genus which has a seasonal occurrence and stimulates an acquired immune response as evaluated by increased WBC production.

### Prevalence and intensity variation between the seasons

In this study we observed a significant difference in the prevalence and intensity of microfilariae between the two seasons. Thus, the dry season was the season in which the prevalence and intensity of goliath frog microfilaria was significantly higher. In fact, prevalence and intensity of filarial worm infestation of amphibian are proportional to the abundance of Culicidae, Psychodidae and ceratopogonidae vectors [22, 23]. Though the wet season is the season during which the insects are more abundant due to the availability of stagnant water on which to oviposit [24, 25]. The results obtained in this study have been different from the previous patterns and the significant high prevalence and intensity observed in goliath frog may be due to their habitat configuration as the goliath frogs live near rivers and waterfalls [26]. During the rainy season, the water flow speed increases and therefore reduces potential breeding sites of larvae and adults that are still flooded whereas, during the dry season, the water flow speed decreases, becoming calm leading to the creation of larval breeding sites along rivers that are important for mosquito breeding as observed in the dry season. On the other hand, it is also possible that during the dry season the frogs are more concentrated in the fewer areas with water, which would also be the breeding sites for the mosquito vector, thus helping to facilitate disease spread.

The intensity of microfilaria infection was positively correlated with the host size and sex. Within the host species, the positive relationship between host size and helminth parasite abundance has been commonly observed for different vertebrate classes [6, 27–29]. Male goliath frogs were found to be significantly infected compared to the females. Sometimes, the weight is also related to age; hence, older frogs would have more time to accumulate infections.

### Influence of infection on haematology and biochemistry

Goliath frog microfilariae caused significant increases in WBC. Other studies in humans and animals infected with gastrointestinal nematodes also revealed higher WBC values [30–32]. In fact, WBC increased in number during the parasites attack indicating a acquired immune response to the parasite.

### Microfilariae genotyping

The successful preliminary mitochondrial genotyping of microfilariae isolated from blood of goliath frog suggests that the same species of filarid infected those 3 frogs coming from different habitats and is consistent with the morphological data set. The phylogenetic analysis, although not exhaustive, showed that the goliath frog’s derived filarial are distinct from all others known genera known to infect humans, mammals and amphibians. Systemic parasite invasions rarely cause a disease in free-living amphibians.

In a stable and healthy natural ecosystem, parasites and their hosts have had the opportunity to coevolve, the parasite causing few pathogenic effects in a healthy animal host. However, if this well-established co-existence is disturbed by, for example, habitat destruction or the indiscriminate movement of animal hosts between habitats; pathogenic effects may become apparent resulting in the destabilisation of the host population [33].

## Conclusion

In conclusion, the microfilariae of goliath frogs may represent a new species of *Icosiella*. The environmental conditions and the habitat play an important role in microfilaria infection dynamics of goliath frog populations. Our future work will focus on finding adult filarial nematodes from these frogs for morphological description and for taxonomic classification. We will also assess the blood parasites of other amphibian species sympatric with goliath frog in order to compare their blood fauna and eventually capture potential vectors of the infections.
